# The differences of lipid profiles between only children and children with siblings: A national survey in China

**DOI:** 10.1038/s41598-018-37695-0

**Published:** 2019-02-05

**Authors:** Li Cai, Bingjie Ma, Lizi Lin, Yajun Chen, Wenhan Yang, Jun Ma, Jin Jing

**Affiliations:** 1 Department of Maternal and Child Health, School of Public Health, Sun Yat-sen University, Guangzhou, China; 2 Department of Maternal and Child Health, School of Public Health, Peking University, Beijing, China; 3 Institute of Child and Adolescent Health, School of Public Health, Peking University, Beijing, China

## Abstract

With the increasing number of the one-child family, it is important to investigate whether the only-child status is associated with dyslipidemia. Among a national sample of 65,347 Chinese children aged 6–17 years, 16,100 lipid profiles were available. Children’s height, weight, total cholesterol (TC), triglycerides (TG), high-density lipoprotein cholesterol (HDL-C), and low-density lipoprotein cholesterol (LDL-C) were measured. In comparison to children with siblings, only children (OC) were more likely to be boys and live in urban areas. OC had less physical activity, less fried food intake, but more meat and dairy intakes. OC had significantly higher levels of TC (3.97 ± 0.78 vs. 3.89 ± 0.77) and LDL-C (2.12 ± 0.65 vs. 2.06 ± 0.64) in the overall group, and also in the subgroups of rural boys and girls. The prevalence of hyper-TC (5.48% vs. 4.43%) and hyper-LDL-C (3.97% vs. 2.96%) were significantly higher in OC than their counterparts. Furthermore, we found higher odds of hyper-LDL-C [1.43 (1.12, 1.83)] in OC after adjustments. In the subgroup analysis, only-child status was associated with increased risk of hyper-TC [1.86 (1.06, 3.26)] and hyper-LDL-C [2.65 (1.14, 6.16)] among rural boys, and hyper-LDL-C among rural girls [2.20 (1.14, 4.22)]. In conclusion, higher levels of TC and LDL-C were found in OC especially for rural children. Being an only-child was associated with increased risk of hyper-LDL-C.

## Introduction

China’s one-child policy had been replaced by a two-child policy in October, 2015^1^, which has drawn attention from both the general public and health professionals. In fact, the one-child rule was introduced in the late 1970s and strictly implemented for urban residents^1^. It was estimated that this policy had created more than 100 million only-child families^2^. These changes in family size largely increased the number of only children (OC) or in other words, first-born children.

Family size and birth order were proposed as key factors affecting children’s health status^3–12^. The effect of birth order on childhood obesity has been well investigated, but with inconsistent results^9–11^. Evidence showed that first-born children had reduced insulin sensitivity^5^, higher blood pressure and risk of developing type 1 diabetes^5–7^ when compared with later-borns. However, the impact of birth order on lipid profiles were inconsistent among previous studies^5,8,12^. Although children without siblings were also first-born in their families, the association between only-child status and their health status was seldom discussed except for childhood obesity. Studies from different countries showed that OC had higher risk for childhood obesity when compared with children with siblings (CWS)^9,13–17^, such as in Sydney [odds ratio (OR) of obesity in OC vs. children with 2 or more siblings = 3.29 (1.78–6.11)]^16^ and in China [OR of obesity/overweight in OC vs. CWS = 4.53 (1.65–12.40)]^17^. More recently, using a sample of 62,444 children, our research group found that OC had a 1.28-fold risk of obesity compared to CWS^18^. These studies suggested that only-child status might play an important role in the risk for cardiovascular disease (CVD) in children.

There are both biological mechanisms and social causation for these associations. It is suggested that the combination of small birth size and catch-up growth in infancy might be the explanations for the birth order effect on CVD^19^. However, the association of birth order or only-child status with CVD risks seem to be largely influenced by social and environmental factors. Competing with and sharing with siblings have considered to be account for personality and behavior differences by birth order^20^.

The doting parents and grandparents tended to feed OC more food, which made them more likely to be over-nourished^21^. Meanwhile, the OC had less opportunities for playing games and certain physical activities with siblings^22^. In modern China, the one-child policy was recognized as a leading contributor to the rising childhood obesity epidemic^17,23,24^. The increasing prevalence of overweight/obesity could result in a much larger population of children with CVD risks, such as abnormal lipid levels^25^. As lipid levels continue to track from childhood into adulthood, improving lipid profiles in children is of great significance for the prevention of adult CVD^26,27^. However, no studies has been conducted to directly investigate the association between only-child status and lipid profiles. A better understanding of lipid profiles in OC, is conducive to an in-depth analysis of the relationship between only-child status and cardiovascular risk factors.

Therefore, using data from a national survey on 16,100 Chinese children, this study aimed to evaluate the differences of lipid profiles between OC and CWS. We also tested whether this association varied by sex and living area.

## Results

The demographic and anthropometric characteristics of the participants are shown in Table 1. A total of 16,100 children aged 6–17 years (mean age: 11.08 years; boys: 50.97%) were included in this study. Compared with CWS, OC were more likely to be boys (53.76% vs. 44.10%, *P* < 0.001) and live in urban areas (65.44% vs. 40.97%, *P* < 0.001). Parents of OC had higher educational levels than their counterparts (*P* < 0.001). OC also had higher height, weight and BMI z-score (all *P* < 0.001). The characteristics of participants by sex and living area are presented in Supplemental Table S1.

**Table 1:** Demographic and anthropometric characteristics of the only children and children with siblings.

Variables	Total (n = 16100)	Only children (n = 11445)	Children with siblings (n = 4655)	*P* value
Age (years)	11.08 ± 3.25	11.15 ± 3.27	10.89 ± 3.20	**0.017**
Sex (%)				
Boys	50.97	53.76	44.10	**<0.001**
Girls	49.03	46.24	55.90	
Living area (%)				
Urban	61.33	65.44	40.97	**<0.001**
Rural	38.76	34.56	59.03	
Paternal educational level (%)				
None/primary	44.78	37.74	64.73	**<0.001**
Secondary	41.09	46.76	29.83	
University or above	14.13	18.50	5.44	
Maternal educational level (%)				
None/primary	48.03	36.82	70.33	**<0.001**
Secondary	39.87	46.81	26.07	
University or above	12.10	16.37	3.60	
Monthly family income (%)				
≤5000 yuan	33.47	31.58	37.97	**<0.001**
5000~12000 yuan	28.68	31.02	23.10	
≥12000 yuan	9.46	10.36	7.32	
N/A	28.39	27.04	31.61	
Height (cm)	147.24 ± 16.92	148.06 ± 17.08	145.23 ± 16.36	**<0.001**
Weight (kg)	41.96 ± 15.73	42.75 ± 16.15	40.01 ± 14.47	**<0.001**
BMI (kg·m^−2^)	18.66 ± 3.81	18.80 ± 3.89	18.33 ± 3.58	**<0.001**
BMI z-score	0.17 ± 1.27	0.20 ± 1.29	0.09 ± 1.21	**<0.001**

BMI, body mass index.

N/A indicates not applicable.

Continuous variables are displayed as mean ± standard deviation.

*P* values are from Mann-Whitney test (continuous variables) and chi-square tests (categorical variables) between only children and children with siblings.

Lifestyles and food intakes between OC and CWS are shown in Supplemental Table S2. Overall, the OC spent less time on MVPA (51.17 ± 0.71 vs. 56.60 ± 1.29, *P* < 0.001) and had less screen time (91.10 ± 1.11 vs. 101.53 ± 1.72, *P* < 0.001). With regard to food intakes, they had higher intakes of vegetables (1.83 ± 0.01 vs. 1.78 ± 0.02, *P* = 0.027), fruits (1.48 ± 0.01 vs. 1.42 ± 0.02, *P* = 0.004), meat products (1.25 ± 0.01 vs. 1.04 ± 0.02, *P* < 0.001), SSBs (0.42 ± 0.01 vs. 0.38 ± 0.01, *P* = 0.009), dairy products (4.55 ± 0.03 vs. 4.05 ± 0.04, *P* < 0.001) and fast food (1.18 ± 0.02 vs. 1.00 ± 0.03, *P* < 0.001), but had less frequency of fried food intake (1.18 ± 0.02 vs. 1.30 ± 0.03, *P* < 0.001). After adjusted for all covariates, we still found that OC devoted less time to sports (*P* < 0.001) but had higher intakes of meat (*P* < 0.001) and dairy products (*P* < 0.001) and less fried food (*P* = 0.002) than CWS. In addition, these differences varied by sex and urbanicity.

As shown in Table 2, the levels of TC (3.97 ± 0.78 vs. 3.89 ± 0.77, *P* < 0.001) and LDL-C (2.12 ± 0.65 vs. 2.06 ± 0.64, *P* < 0.001) were higher among OC when compared with that in CWS. To further analyze the influence of sex and urban-rural differences on lipid profiles, we divided the subjects into four subgroups. In urban areas, the only-sons (3.96 ± 0.81 vs. 3.91 ± 0.81, *P* = 0.036) and only-daughters (4.04 ± 0.80 vs. 3.99 ± 0.84, *P* = 0.025) showed higher levels of TC than their counterparts. There were no significant differences in TG, LDL-C, and HDL-C between OC and CWS living in urban areas. In rural areas, the only-sons showed higher levels of TC (3.81 ± 0.71 vs. 3.73 ± 0.68, *P* = 0.005), TG (0.89 ± 0.48 vs. 0.83 ± 0.38, *P* = 0.001) and LDL-C (1.97 ± 0.58 vs. 1.91 ± 0.54, *P* = 0.008) than boys with sibling. On the other hand, the only-daughters living in rural areas showed higher levels of TC (4.05 ± 0.71 vs. 3.86 ± 0.66, *P* < 0.001), LDL-C (2.10 ± 0.60 vs. 1.97 ± 0.54, *P* < 0.001) and HDL-C (1.39 ± 0.31 vs. 1.35 ± 0.31, *P* = 0.003) than their counterparts. When adjusted for all covariates, we only found differences in levels of TC and LDL-C between OC and CWS among the overall group and those who lived in rural areas, including both boys and girls (all adjusted *P* < 0.05).

**Table 2:** Lipid profiles of the only children and children with siblings by sex and living area.

Variables	Total	Urban (n = 9874)		Rural (n = 6226)	
		Boys (n = 5082)	Girls (n = 4792)	Boys (n = 3124)	Girls (n = 3102)
Sample size					
Only children	11445	3885	3371	2268	1921
Children with siblings	4655	1197	1421	856	1181
TC (mmol/L)					
Only children	3.97 ± 0.78	3.96 ± 0.81	4.04 ± 0.80	3.81 ± 0.71	4.05 ± 0.71
Children with siblings	3.89 ± 0.77	3.91 ± 0.81	3.99 ± 0.84	3.73 ± 0.68	3.86 ± 0.66
*P* value	**<0.001**	**0.036**	**0.025**	**0.005**	**<0.001**
Adjusted P* value	**0.028**	0.181	0.941	**0.004**	**0.001**
TG (mmol/L)					
Only children	0.92 ± 0.46	0.89 ± 0.42	0.95 ± 0.44	0.89 ± 0.48	0.97 ± 0.50
Children with siblings	0.82 ± 0.43	0.88 ± 0.42	0.96 ± 0.43	0.83 ± 0.38	0.96 ± 0.45
*P* value	0.763	0.688	0.548	**0.001**	0.463
Adjusted P* value	0.147	0.464	0.707	0.200	0.167
LDL-C (mmol/L)					
Only children	2.12 ± 0.65	2.15 ± 0.67	2.21 ± 0.67	1.97 ± 0.58	2.10 ± 0.60
Children with siblings	2.06 ± 0.64	2.11 ± 0.65	2.19 ± 0.72	1.91 ± 0.54	1.97 ± 0.54
*P* value	**<0.001**	0.059	0.458	**0.008**	**<0.001**
Adjusted P* value	**0.008**	0.262	0.712	**0.016**	**0.008**
HDL-C (mmol/L)					
Only children	1.36 ± 0.34	1.37 ± 0.36	1.37 ± 0.35	1.33 ± 0.31	1.39 ± 0.31
Children with siblings	1.36 ± 0.34	1.37 ± 0.35	1.36 ± 0.35	1.35 ± 0.31	1.35 ± 0.31
*P* value	0.383	0.742	0.284	0.064	**0.003**
Adjusted P* value	0.494	0.167	0.315	0.787	0.102

TC, total cholesterol; TG, triglycerides; LDL-C, low-density lipoprotein cholesterol; HDL-C, high-density lipoprotein cholesterol.

Continuous variables are displayed as mean ± standard deviation.

The *P* value was compared with only children and children with siblings.

In the subgroups, P* value was adjusted for age, parental educational levels, monthly family incomes, BMI z-score, MVPA time, screen time and food intakes and a random effect for provinces; in the total group, *P value was additionally adjusted for sex and living areas.

**Figure 1:**
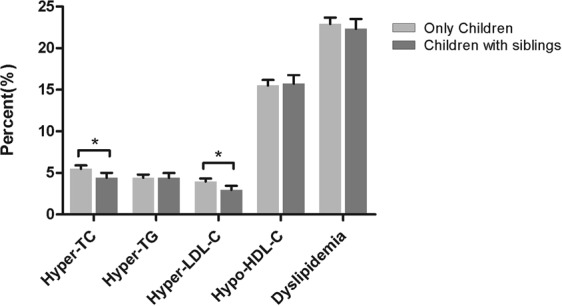
Prevalence of dyslipidemia between only children and children with siblings. *P < 0.05. Dyslipidemia refers to at least one of the following: hyper-TC, hyper-TG, hyper-LDL-C, and hypo-HDL-C.

**Figure 2:**
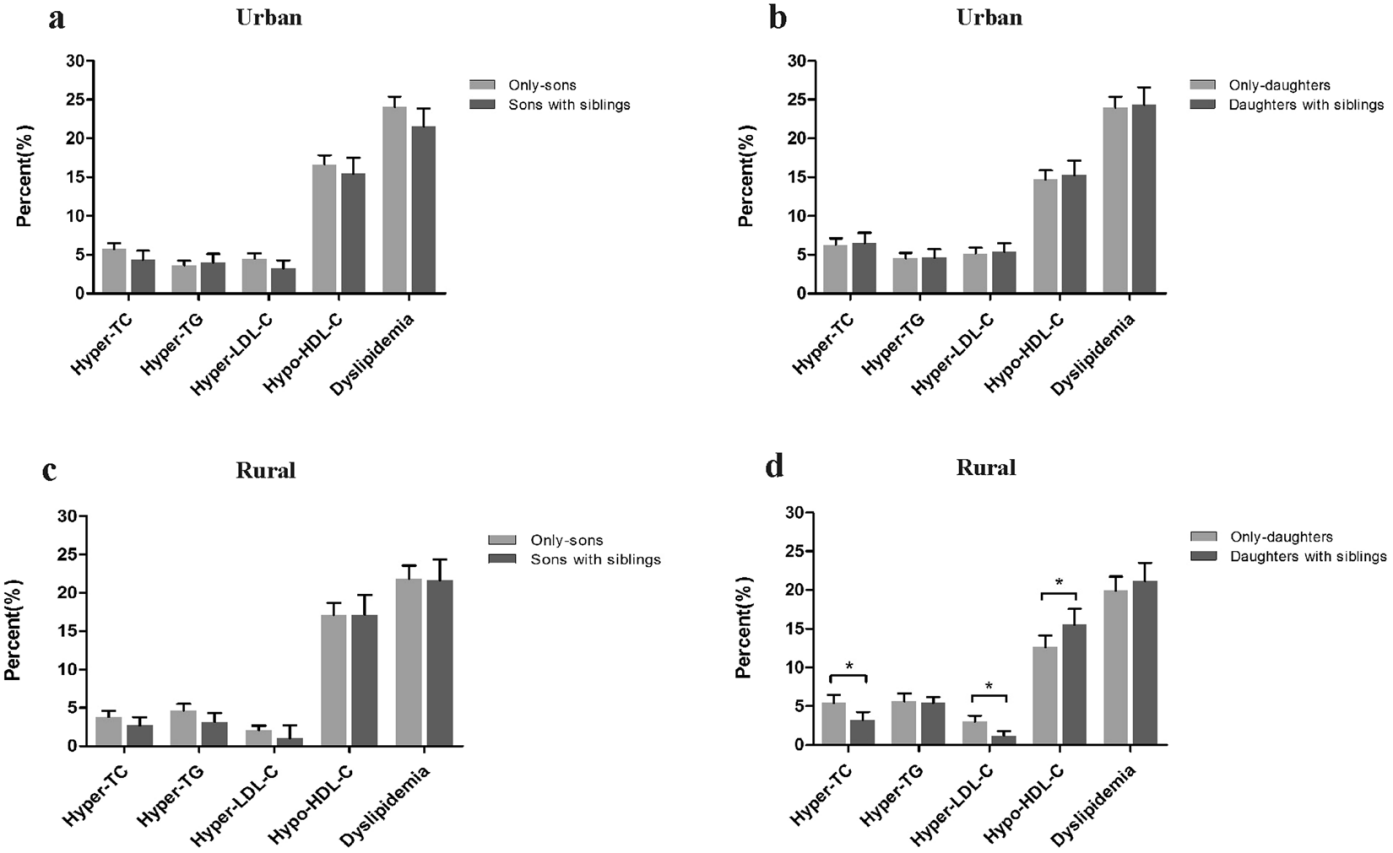
Prevalence of dyslipidemia between only children and children with siblings by sex and living area. *P < 0.05. Dyslipidemia refers to at least one of the following: hyper-TC, hyper-TG, hyper-LDL-C, and hypo-HDL-C.

Figure 1 shows the prevalence of various types of dyslipidemia. The prevalence of hyper-TC (5.48% vs. 4.43%, *P* = 0.006) and hyper-LDL-C (3.97% vs. 2.96%, *P* = 0.002) were higher in OC than that in CWS. There was no significant difference in the prevalence of hyper-TG, hypo-HDL-C, and dyslipidemia between OC and CWS. Results from subgroup analysis by sex and living area are presented in Figure 2. Higher prevalence of hyper-TC (5.47% vs. 3.22%, *P* = 0.004), hyper-LDL-C (3.02% vs. 1.19%, *P* = 0.001), and lower prevalence of hypo-HDL-C (12.65% vs. 15.50%, *P* = 0.025) were observed only among only-daughters living in rural areas. The associations between the only-child status and dyslipidemia were also examined by generalized linear mixed models (see Table 3). After adjustment for covariates, we found higher odds of hyper-LDL-C (OR: 1.43; 95% CI: 1.12–1.83) in OC. In the subgroup analysis, we found no significant associations between only-child status and dyslipidemia among urban children. However, only-child status was associated with increased risk of hyper-TC (OR: 1.86; 95% CI: 1.06–3.26) and hyper-LDL-C (OR: 2.65; 95% CI: 1.14–6.16) among rural boys, and hyper-LDL-C among rural girls (OR: 2.20; 95% CI: 1.14–4.22).

**Table 3:** Odds ratios of only-child status for dyslipidemia according to generalized linear mixed models.

	Total		Urban				Rural			
			Boys		Girls		Boys		Girls	
	OR (95%CI)	*P*	OR (95%CI)	*P*	OR (95%CI)	*P*	OR (95%CI)	*P*	OR (95%CI)	*P*
Hyper-TC										
Model 1	1.12 (0.94, 1.34)	0.197	1.22 (0.87, 1.71)	0.246	0.86 (0.64, 1.15)	0.305	1.50 (0.92, 2.45)	0.108	1.23 (0.81, 1.86)	0.342
Model 2	1.15 (0.95, 1.39)	0.149	1.14 (0.80, 1.62)	0.474	0.97 (0.70, 1.33)	0.843	**1.86 (1.06, 3.26)**	**0.030**	1.15 (0.74, 1.78)	0.542
Model 3	1.15 (0.95, 1.40)	0.150	1.14 (0.80, 1.62)	0.470	0.97 (0.70, 1.34)	0.850	**1.86 (1.06, 3.26)**	**0.030**	1.15 (0.74, 1.78)	0.540
Hyper-TG										
Model 1	1.05 (0.88, 1.27)	0.580	0.92 (0.64, 1.34)	0.678	0.85 (0.61, 1.20)	0.359	1.41 (0.90, 2.20)	0.137	1.06 (0.75, 1.51)	0.729
Model 2	1.14 (0.93, 1.40)	0.195	1.17 (0.77, 1.78)	0.460	0.94 (0.63, 1.41)	0.760	1.42 (0.88, 2.29)	0.148	1.12 (0.77, 1.63)	0.549
Model 3	1.14 (0.93, 1.40)	0.197	1.18 (0.78, 1.80)	0.430	0.94 (0.63, 1.41)	0.759	1.43 (0.89, 2.30)	0.144	1.12 (0.77, 1.63)	0.552
Hyper-LDL-C										
Model 1	**1.27 (1.03, 1.58)**	**0.028**	1.29 (0.88, 1.91)	0.197	0.94 (0.68, 1.29)	0.690	**2.37 (1.12, 5.01)**	**0.024**	**2.22 (1.18, 4.17)**	**0.013**
Model 2	**1.44 (1.13, 1.83)**	**0.004**	1.40 (0.90, 2.17)	0.131	1.10 (0.76, 1.60)	0.600	**2.65 (1.14, 6.16)**	**0.024**	**2.19 (1.14, 4.23)**	**0.019**
Model 3	**1.43 (1.12, 1.83)**	**0.004**	1.41 (0.91, 2.19)	0.127	1.11 (0.76, 1.60)	0.596	**2.65 (1.14, 6.16)**	**0.024**	**2.20 (1.14, 4.22)**	**0.018**
Hypo-HDL-C										
Model 1	0.99 (0.89, 1.10)	0.816	1.00 (0.81, 1.23)	0.985	0.84 (0.69, 1.04)	0.114	1.04 (0.83, 1.30)	0.763	0.97 (0.77, 1.22)	0.798
Model 2	1.00 (0.89, 1.12)	0.968	1.01 (0.80, 1.28)	0.904	0.90 (0.71, 1.14)	0.386	1.02 (0.81, 1.30)	0.842	0.96 (0.75, 1.23)	0.735
Model 3	1.00 (0.89, 1.12)	0.968	1.01 (0.80, 1.28)	0.901	0.90 (0.71, 1.14)	0.387	1.02 (0.81, 1.30)	0.841	0.96 (0.75, 1.23)	0.739
Dyslipidemia										
Model 1	1.00 (0.91, 1.10)	0.968	1.04 (0.87, 1.25)	0.675	0.87 (0.73, 1.03)	0.110	1.03 (0.84, 1.27)	0.744	0.97 (0.80, 1.19)	0.788
Model 2	1.02 (0.92, 1.13)	0.724	1.08 (0.89, 1.32)	0.445	0.93 (0.76, 1.13)	0.457	1.03 (0.83, 1.28)	0.783	0.96 (0.78, 1.18)	0.689
Model 3	1.02 (0.92, 1.13)	0.730	1.09 (0.89, 1.33)	0.423	0.93 (0.76, 1.13)	0.461	1.03 (0.83, 1.28)	0.790	0.96 (0.78, 1.18)	0.692

OR, odds ratio; CI, confidence interval; TC, total cholesterol; TG, triglycerides; LDL-C, low-density lipoprotein cholesterol; HDL-C, high-density lipoprotein cholesterol; OC, only children; CWS, children with siblings.

Dyslipidemia refers to at least one of the following: hyper-TC, hyper-TG, hyper-LDL-C, and hypo-HDL-C.

In the subgroups, model 1 was adjusted for age, parental educational levels, monthly family incomes and a random effect for provinces; model 2 was adjusted for variables in model 1, MVPA time, screen time, and food intakes; model 3 was adjusted for variables in model 2 and BMI z-score. In the total group, model 1 was additionally adjusted for sex and living areas.

## Discussion

Using national data of 16,100 children, we found that OC had higher levels of TC and LDL-C than CWS, especially in rural China. In addition, being an only child was associated with increased risk of hyper-LDL-C especially among rural children.

The impact of family characteristic (e.g. birth order) on lipid profiles has been examined in previous studies^5,8,12^. It was hypothesised that first-borns may be at greater risk of CVD than their later born siblings^28^. A cohort study found that the first-borns showed significantly higher levels of TC and LDL-C in early adulthood^8^. In our study, we found that OC showed less favourable lipid profiles than their counterparts. To some extent, there may be some similar underlying mechanisms of higher metabolic risks in both OC and first-borns. While two studies found that there were no impact of birth order on children’s lipid profiles, and one of them found no differences in stature between first-borns with or without siblings^5,12^. These two studies, however, had a very small sample size (312 and 85, respectively), and they didn’t take into account the influence of behavioral and environmental factors.

Two tentative explanations could be offered to explain that OC showed elevated lipid levels. First, OC were all first-borns. There was evidence that the in-utero growth of first-born babies might be restrained and they had lower birth weight and accelerated early catch-up growth^28,29^, both of which were independent risk factors for metabolic and cardiovascular diseases^19,30–33^. Second, their higher metabolic and cardiovascular risk may be partly resulted from the behavioral or environmental changes. It was suggested that the focus of attention and resources from OC’s families might lead to over-feeding^23^. In our study, OC tended to have higher intakes of meat and dairy products. On the other hand, we found that OC had less PA time than CWS, although no significant difference was found in screen time. Siblings played an important role in interactions, activities and cooperative play, leading to more PA time in CWS^21^. The combination of higher food intake and reduction in PA might result in energy imbalance in OC and therefore lead to higher risk of obesity and other metabolic diseases^23,25^.

We also found higher prevalence of hyper-TC in OC, however, the difference became insignificant after adjustment for parental educational levels and family incomes. This suggested that the association between the only-child status and dyslipidemia may be partly dependent on these factors. It was suggested that triglycerides level was inversely associated with education and salary in some developed areas^34,35^. However, other studies found that individuals of lower socioeconomic status (SES) had lower levels of TC and LDL-C in developing areas^36,37^. In our study, only-child families were characterized as higher in SES and OC had higher levels of TC and LDL-C, which were consistent with findings in developing areas, indicating greater CVD risks among OC. This phenomenon may be partly explained by that higher income levels was associated with higher consumption of high-fat, high-energy food in modern China^24^.

The differences of lipid profiles between OC and CWS were less pronounced in urban China, which might be relate to the similar parental attention and family resources between OC and CWS in urban areas. China’s economy has developed rapidly during the past decades, especially in urban areas. Urban children had better health and nutritional status than rural children in China^38^. Under the low fertility regime, fewer children in each household might be provided similar attention and resources from their parents.

Another important result of this study was that OC in rural China had a more than double risk of having hyper-LDL-C compared to rural CWS. In fact, those rural parents of OC showed higher socio-economic and educational levels than the rural parents of CWS (Supplemental Table S1). In terms of health-related behaviors, rural OC had less MVPA time but higher intakes of meat and dairy products compared to rural CWS. These phenomena may provide some ideas to explain our finding, however, the underlying mechanism remains to be determined. Considering the abovementioned urban-rural differences, it is important to pay additional attention to blood lipid profiles in OC who live in rural areas.

The fertility rates have been declining throughout the world during the recent decades, especially in Europe and many Asian countries^39,40^. Globally, the total fertility rates declined from 4.984 children per woman in 1960 to 2.451 in 2015^41^. As a result, there has been a great increase in the number of one-child families. The health of the only/first-born children will be a worldwide public health challenge. China’s one-child policy has resulted in a large number of one-child family, which provides us a context to study the related characteristics and brings the world a warning about the higher risks of metabolic and cardiovascular diseases in OC.

This study had several strengths. First, to our knowledge, this was the first national study to explore the association between only-child status and dyslipidemia. Second, this study considered both sex and urban-rural disparities. Although the one-child rule was enforced for both urban and rural residents in China, this rule was virtually unenforceable in rural areas^1^. Furthermore, there has been a huge urban–rural disparity in many ways, including social economic status^42^, utilization^43^, dietary pattern and nutrition status^38^. In this study, the differences of TC and LDL-C levels between OC and CWS were particularly apparent in rural China. The only-child status was associated with increased risk of hyper-LDL-C among rural children. These results suggested that the only-child status may be a risk factor of dyslipidemia in rural children. Thus, it is better to take urban-rural disparities into account in the intervention programs for the childhood dyslipidemia.

Limitations in this study should be highlighted. First, the causality of the relationships observed cannot be inferred because of the cross-sectional design. Second, there were some factors that we did not take into account, such as food preparation practices, health status of parents, parent-child interaction, etc. Third, self-report bias in lifestyle and socio-economic factors could not be excluded. Fourth, the brief self-reported dietary assessment was subjected to recall bias with not very high ICC. Fifth, there were significant differences in age between participants who were included in the analysis and those who were not. Nevertheless, we confirmed that the association between only-child status and lipid profiles was similar in different age group.

## Conclusions

Higher levels of TC and LDL-C were found in OC especially for those who lived in rural areas. Being an only-child was associated with increased risk of hyper-LDL-C especially among rural children. Early prevention of childhood dyslipidemia in OC especially in Chinese rural areas is urgent needed. Furthermore, the urban-rural disparity of lipid profiles between OC and CWS should be well considered in the preventive guidelines and public health policies.

## Methods

### Design and Study Subjects

This study was based on the baseline data of a multi-centered school-based obesity intervention program (NCT02343588)^44^. The baseline survey was conducted between September 2013 and November 2013. Using a multistage cluster sampling design, 94 schools were selected from 7 provinces/regions, which included Liaoning (northeast), Tianjin (north), Ningxia (northwest), Shanghai (east), Chongqing (west), Hunan (central), and Guangdong (south). Invitation letters, information sheets and a presentation containing study details were sent to the principals of selected schools. With the principal’s permission, all students in the selected schools were invited to participate in the survey and a total of 65,347 children aged 6 to 17 years were recruited. Furthermore, a subsample was selected for blood collection. In brief, half of the schools in each province/region were randomly chosen, and half of the classes in each grade were selected from these schools. In each selected class, all the students were invited to take blood sampling. Children who had missing data on age, gender, only-child status, or living areas (total n = 707) were excluded. Finally, 16,100 lipid profiles were available in this study. Comparing to the overall sample, the subsample (n = 16, 100) was similar in sex distribution (boys: 50.97% vs. 51.60%, *P* > 0.05), but was slightly older (11.08 ± 3.25 years vs. 10.82 ± 3.30 years, *P* < 0.05).

The study was approved by the Ethical Committee of the Peking University and was performed in accordance with principles in the Declaration of Helsinki. Written informed consents were obtained from all participating students and their parents.

### Anthropometric Measurements

Children’s height and weight were measured by qualified technicians, with the child in light clothing without shoes. Body weight was measured to the nearest 0.1 kg, and height was measured to the nearest mm. Each subject’s height and weight were measured twice and we calculated the average values. Body mass index (BMI) was expressed by the body weight (in kilograms) divided by height (in meters) squared and then transformed to a BMI z-score based on data from the sex- and age-specific World Health Organization Growth reference (5–19 years)^45^.

### Questionnaire Assessment

The self-reported questionnaires were designed to collect information on socio-demographic factors and other behavioral factors. Children and families’ basic demographic information including age, sex, only child or not, living in urban or rural areas, provinces, parental educational levels, and monthly family incomes were filled out by parents. Children’s health-related behaviors including physical activities (PA), screen time (television time and computer time), and food intakes were completed by parents and children together. Participants were asked about daily consumptions of vegetables, fruits, sugar-sweetened beverages (SSBs) and meat products, and weekly frequencies of having dairy products, high-energy food, fried food, and monthly frequency of having western fast food. Participants were asked about the frequency and time (hours and minutes) they spent daily on screen time, vigorous-intensity physical activities (VPA), and moderate-intensity physical activities (MPA) over the past 7 days, respectively. VPA and MPA were defined according to the International Physical Activity Questionnaire (IPAQ)^46^. Moderate to vigorous physical activities (MVPA) time was calculated as the sum of VPA and MPA time. In addition, a quartile method was used to class and code the intake of each food item for each child (1: bottom 25%, 2: 25%-50%, 3: 50%-75%, and 4: top 25%). The total food intake was calculated as the sum of the total scores and was quartered into four groups. The reliability and validity of the questionnaires of sedentary behaviors, PA, and dietary intakes were assessed in a sample of 298 primary school students in Guangzhou, China. Generally, the reliability coefficient of 0.40–0.75 and correlation coefficient of 0.20–0.60 are considered acceptable^47,48^. Our results indicate that the questionnaires have acceptable reliability and validity (e.g. the average Intra-class Correlation Coefficient (ICC) of PA was 0.460, and the average Spearman’s Correlation Coefficient (SCC) of PA was 0.407, both *P* < 0.05).

### Blood Lipid Profiles

After at least 12 hours of overnight fasting, venous blood specimens (5 ml) were collected into ethylenediaminetetraacetic acid (EDTA) vacuum tubes, for the measurement of serum lipid levels. The blood specimens were separated by centrifugation at 3000r for 15 minutes, aliquoted and stored at −80 °C until testing. Serum lipid levels, including total cholesterol (TC), triglycerides (TG), high-density lipoprotein cholesterol (HDL-C) and low-density lipoprotein cholesterol (LDL-C) were assessed at a biomedical analysis company, which was accredited by Peking University. TC and TG were measured by enzymatic methods; LDL-C and HDL-C were measured by clearance method.

Serum lipids were categorized by using the Chinese criteria for dyslipidemia in children and adolescents^49^: high TC, ≥200 mg/dL (5.20 mmol/L); high TG, ≥150 mg/dL (1.76 mmol/L); high LDL-C, ≥130 mg/dL (3.38 mmol/L); and low HDL-C, ≤40 mg/dL (1.04 mmol/L).

### Statistical Analysis

Statistical analysis was performed using SPSS 22.0. Descriptive statistics were used to characterize the population. Continuous variables were presented as mean values and standard errors or deviations, while categorical variables were presented as proportions. Statistically significant differences of continuous variables with non-normal distribution (age, height, weight, BMI, BMI z-score, TC, TG, LDL-C, and HDL-C) and categorical variables between OC and CWS were determined using Mann-Whitney test and chi-square test, respectively. Linear mixed models were used in the comparison of lipid profiles between OC and CWS; and generalized linear mixed models were used to assess the association between the only-child status and dyslipidemia. All models were adjusted for age, sex, living areas, parental educational levels, monthly family incomes, BMI z-score, MVPA time, screen time, food intakes, and a random effect for provinces. In addition, we conducted stratified analysis by child’s sex and urban/rural residence to further explore the related differences by these factors. A two-sided *P* value < 0.05 was considered significant.

## Supplementary information

Supplemental Table S1,Supplemental Table S2.

## Data Availability

The datasets generated during and/or analysed during the current study are available from the corresponding author on reasonable request.
