# Supplementary material for: The differences of lipid profiles between only children and children with siblings: A national survey in China
Source: Sci Rep. 2019 Feb 5;9:1441. doi: 10.1038/s41598-018-37695-0 (PMC6363803; doi:10.1038/s41598-018-37695-0)
Supplement: Supplementary file 1 — Supplemental Table S1,Supplemental Table S2. [file 41598_2018_37695_MOESM1_ESM.pdf]

The differences of lipid profiles between only children and children with siblings: A national survey in China

Li Cai[#] [1], Bingjie Ma[#] [1], Lizi Lin[1, 2], Yajun Chen[1], Wenhan Yang[1], Jun Ma* [3], Jin Jing* [1]

Address: [1]Department of Maternal and Child Health, School of Public Health, Sun Yat-sen University, Guangzhou, China; [2]Department of Maternal and Child Health, School of Public Health, Peking University, Beijing, China and [3]Institute of Child and Adolescent Health, School of Public Health, Peking University, Beijing, China.

Email: Li Cai - caili5@mail.sysu.edu.cn;

Bingjie Ma - mabj@mail2.sysu.edu.cn;

Lizi Lin - linlizi@bjmu.edu.cn;

Yajun Chen - chenyj68@mail.sysu.edu.cn;

Wenhan Yang - yangwhan@mail.sysu.edu.cn;

Jun Ma -majunt@bjmu.edu.cn;

Jin Jing - jingjin@mail.sysu.edu.cn

[#] Equal contributors

* Correspondence author

Supplemental Table S1 Demographic and anthropometric characteristics of the only children and children with siblings by sex and by living area

Variables	Urban(n=9874)						Rural(n=6226)					
	Boys(n=5082)			Girls(n=4792)			Boys(n=3124)			Girls(n=3102)		
	Only children	Children with siblings	*P*	Only children	Children with siblings	*P*	Only children	Children with siblings	*P*	Only children	Children with siblings	*P*
Sample size	3885	1197		3371	1421		2268	856		1921	1181	
Age (years)	10.83±3.02	10.59±2.88	**0.017**	10.81±3.09	10.78±3.10	0.773	11.78±3.46	10.87±3.40	**<0.001**	11.67±3.64	11.36±3.40	**0.019**
Paternal educational level (%)												
None/primary	25.60	56.44	**<0.001**	18.80	53.82	**<0.001**	53.41	73.99	**<0.001**	48.46	78.80	**<0.001**
Secondary	51.58	37.04		54.72	38.18		36.67	22.43		40.75	18.45	
University or above	22.83	6.53		26.48	8.01		10.22	3.58		10.79	2.75	
Maternal educational level (%)												
None/primary	26.64	62.65	**<0.001**	20.23	58.57	**<0.001**	55.37	83.41	**<0.001**	52.68	81.90	**<0.001**
Secondary	53.71	32.57		56.41	36.51		34.85	14.32		37.30	16.21	
University or above	19.65	4.78		23.36	4.92		9.42	2.27		10.01	1.89	
Monthly family income (%)												
≤5000 yuan	30.63	32.85	**0.001**	29.68	32.53	**<0.001**	34.13	40.08	**<0.001**	33.20	46.31	**<0.001**
5000~12000 yuan	31.50	24.31		32.88	26.13		28.54	22.22		30.17	19.60	
≥12000 yuan	10.61	11.65		12.07	9.87		8.95	5.03		8.90	2.73	
N/A	27.26	31.19		25.37	31.37		28.38	32.67		27.73	31.36	
Height (cm)	148.09±17.73	145.53±17.42	**<0.001**	145.59±14.95	143.71±15.20	**<0.001**	152.71±18.99	146.65±18.16	**<0.001**	146.81±15.73	145.70±15.05	**0.049**
Weight (kg)	42.98±16.63	39.86±14.36	**<0.001**	39.58±13.29	38.28±13.01	**0.002**	47.70±18.88	41.70±16.79	**<0.001**	42.03±14.73	41.02±14.21	0.059
BMI ($kg \cdot m^{-2}$)	18.87±3.95	18.21±3.41	**<0.001**	18.12±1.47	17.96±3.29	0.135	19.62±4.26	18.63±3.91	**<0.001**	18.85±3.79	18.68±3.77	0.227
BMI z-score	0.36±1.41	0.20±1.32	**0.001**	-0.01±1.15	-0.05±1.08	0.295	0.39±1.36	0.24±1.37	**0.007**	0.06±1.11	0.06±1.10	0.956

BMI, body mass index.

N/A indicates not applicable.

Continuous variables are displayed as mean ± standard deviation.

P values are from Mann-Whitney test (continuous variables) and chi-square tests (categorical variables) between only children and children with siblings.

Supplemental Table S2 Lifestyles and eating habits between only children and children with siblings by sex and by living area

Health-related behaviors	Total	Urban(n=9874)		Rural(n=6226)	
		Boys(n=5082)	Girls(n=4792)	Boys(n=3124)	Girls(n=3102)
Health-related lifestyle					
Screen time (min/day)					
Only children	91.10±1.11	95.63±1.99	75.62±1.69	109.85±2.73	83.63±2.49
Children with siblings	101.53±1.72	115.25±3.68	96.94±3.04	106.58±4.01	89.85±3.12
P value	**<0.001**	**<0.001**	**<0.001**	0.518	0.120
Adjusted *P** value	0.594	0.060	**0.007**	0.218	0.392
MVPA time (min/day)					
Only children	51.17±0.71	54.36±1.25	39.97±0.99	63.91±1.86	47.60±1.62
Children with siblings	56.60±1.29	59.41±2.21	44.79±1.90	70.64±3.56	58.57±2.91
P value	**<0.001**	**0.039**	**0.013**	**0.042**	**<0.001**
Adjusted *P** value	**<0.001**	0.112	**<0.001**	**0.030**	**0.010**
Dietary intake					
Vegetables (servings [a]/day)					
Only children	1.83±0.01	1.90±0.03	1.89±0.03	1.78±0.03	1.71±0.03
Children with siblings	1.78±0.02	1.85±0.05	1.77±0.03	1.73±0.48	1.74±0.04
P value	**0.027**	0.370	0.070	0.354	0.453
Adjusted *P** value	0.621	0.838	0.098	0.381	0.257
Fruits (servings [a]/day)					
Only children	1.48±0.01	1.51±0.02	1.56±0.02	1.38±0.03	1.44±0.03
Children with siblings	1.42±0.02	1.43±0.04	1.25±0.03	1.32±0.04	1.43±0.03
P value	**0.004**	0.124	0.077	0.229	0.831
Adjusted *P** value	0.485	0.993	0.757	0.399	0.449
Meat products (serving [b]/day)					
Only children	1.25±0.01	1.44±0.03	1.12±0.02	1.30±0.03	1.07±0.03
Children with siblings	1.04±0.02	1.27±0.04	1.03±0.03	1.07±0.04	0.82±0.03
P value	**<0.001**	**<0.001**	**0.009**	**<0.001**	**<0.001**
Adjusted *P** value	**<0.001**	0.214	0.839	**0.003**	**<0.001**
SSBs (cup [c]/day)					
Only children	0.42±0.01	0.47±0.02	0.30±0.01	0.57±0.02	0.33±0.01
Children with siblings	0.38±0.01	0.47±0.03	0.32±0.02	0.46±0.03	0.30±0.02
P value	**0.009**	0.944	**0.042**	0.053	0.191
Adjusted *P** value	0.682	0.543	0.714	0.500	0.253
Diet behaviors					

Dairy products (day/week)					
Only children	4.55±0.03	4.58±0.05	4.59±0.05	4.41±0.06	4.61±0.06
Children with siblings	4.05±0.04	4.10±0.08	4.03±0.07	3.98±0.10	4.05±0.08
P value	**<0.001**	**<0.001**	**<0.001**	**<0.001**	**<0.001**
Adjusted *P** value	**<0.001**	**0.004**	**0.036**	0.231	0.065
High-energy snacks (time/week)					
Only children	2.02±0.02	1.90±0.04	2.02±0.04	2.07±0.05	2.16±0.05
Children with siblings	2.07±0.03	1.92±0.06	2.23±0.06	1.89±0.07	2.16±0.05
P value	0.197	0.777	**0.002**	**0.030**	0.927
Adjusted *P** value	0.731	0.263	0.364	0.221	0.104
Fried food (time/week)					
Only children	1.18±0.02	1.14±0.03	1.05±0.03	1.39±0.04	1.20±0.04
Children with siblings	1.30±0.03	1.22±0.05	1.14±0.04	1.46±0.06	1.45±0.05
P value	**<0.001**	**0.019**	**0.039**	0.319	**<0.001**
Adjusted *P** value	**0.002**	0.543	0.838	0.057	**<0.001**
Western fast food (time/month)					
Only children	1.18±0.02	1.23±0.04	1.18±0.04	1.19±0.05	1.10±0.04
Children with siblings	1.00±0.03	1.07±0.06	1.11±0.06	0.98±0.08	0.84±0.05
P value	**<0.001**	0.051	0.270	**0.034**	**<0.001**
Adjusted *P** value	0.268	0.825	0.885	0.870	**0.010**

MVPA, moderate-to-vigorous physical activity; SSBs, sugar-sweetened beverages.

Continuous variables are displayed as mean ± standard error.

The *P* value was compared with only children and children with siblings.

[a] A serving of fruit or vegetable is equivalent to 100 g. [b] A serving of meat products is equivalent to 75 g. [c] A cup is equivalent to 250 ml.

In the subgroups, *P** value was adjusted for age, parental educational levels, monthly family incomes, BMI z-score, MVPA time, screen time and food intakes and a random effect for provinces; in the total group, *P** value was additionally adjusted for sex and living areas.
